# On the interpretability of predictors in spatial data science: the information horizon

**DOI:** 10.1038/s41598-020-73773-y

**Published:** 2020-10-07

**Authors:** Thorsten Behrens, Raphael A. Viscarra Rossel

**Affiliations:** 1 Cluster of Excellence “Machine Learning: New Perspectives for Science”, Eberhard Karls University Tuebingen, Maria von Linden Str. 6, 72076 Tübingen, Germany; 2 Soil and Spatial Data Science, Soilution GbR, Heiligegeiststrasse 13, 06484 Quedlinburg, Germany; 3 Soil and Landscape Science, School of Molecular and Life Sciences, Faculty of Science and Engineering, Curtin University, GPO Box U1987, Perth, WA Australia

**Keywords:** Environmental sciences, Ecological modelling

## Abstract

Two important theories in spatial modelling relate to structural and spatial dependence. Structural dependence refers to environmental state-factor models, where an environmental property is modelled as a function of the states and interactions of environmental predictors, such as climate, parent material or relief. Commonly, the functions are regression or supervised classification algorithms. Spatial dependence is present in most environmental properties and forms the basis for spatial interpolation and geostatistics. In machine learning, modelling with geographic coordinates or Euclidean distance fields, which resemble linear variograms with infinite ranges, can produce similar interpolations. Interpolations do not lend themselves to causal interpretations. Conversely, with structural modeling, one can, potentially, extract knowledge from the modelling. Two important characteristics of such interpretable environmental modelling are scale and information content. Scale is relevant because very coarse scale predictors can show nearly infinite ranges, falling out of what we call the information horizon, i.e. interpretation using domain knowledge isn’t possible. Regarding information content, recent studies have shown that meaningless predictors, such as paintings or photographs of faces, can be used for spatial environmental modelling of ecological and soil properties, with accurate evaluation statistics. Here, we examine under which conditions modelling with such predictors can lead to accurate statistics and whether an information horizon can be derived for scale and information content.

## Introduction

It is well known that correlation does not signify causality and that domain experts should interpret with care models and their feature importance. In this respect explainable artificial intelligence or interpretable machine learning is defined as the extraction of relevant knowledge about domain relationships contained in the data, or as descriptive accuracy, which also depends on accurate model predictions, i.e. predictive accuracy^1^. This study pertains to the interpretability of spatial environmental predictors used in spatial modelling with machine leaning.

Recent publications have demonstrated that obviously meaningless predictors, e.g. paintings or photographs of faces, can produce spatial model of species distribution or soil properties that validate well and with meaningless but apparently important predictors^2,3^. We define ’meaningless’ as (i) not structurally related or not based on domain relationships, (ii) not spatially related, and (iii) completely unrelated. Here, we aim to explain the mechanisms that lead to the detection of patterns or trends from such meaningless data. We focus (i) on the conditions under which such meaningless predictors can produce accurate evaluation statistics and (ii) on the question under which conditions structural relationships can lead to useful interpretations.

Recently, we showed that the range of the variogram is related to the maximum useful range for spatial contextual structural modeling^4^. This relevant range of scales hints at why using paintings or photographs of faces as predictors can, under certain conditions, describe the systematic variation in soil or other environmental properties. The rationale is that the range of spatial dependence exhibited in these photographs or paintings, is to some degree, similar to the range of the environmental property (i.e. the response variable). Conversely, if photographs of finely textured fabrics with shorter ranges or smaller scales would have been used, the modelling would not have worked. Thus, our first hypothesis is:

*In spatial modelling with machine learning, using a sufficient number of meaningless (or structurally independent) predictors, with a similar range (or similar scale) to that of the response variable will produce accurate model evaluation statistics.*

This has further implications for spatial modelling using Euclidean distance fields (EDF^5^, see “Reference models” section) as predictors. EDFs can be represented by linear variograms with infinite ranges. They can be used as predictors for spatial modelling with any machine learning algorithm, resembling spatial interpolations and can help to account for non-stationarities in hybrid models^5^. However, models derived with EDFs cannot be interpreted in terms of structural relationships between the response variable and the predictors. They can only help to reveal relative spatial relationships, which is similar for Kriging interpolations^6^ or Spatial Filtering^7^. Therefore, our second hypothesis is:

*Spatial modeling using a sufficient number predictors (of any kind) with similar or longer ranges (or with coarser scales) than the response variable will produce accurate evaluation statistics, no matter how long the ranges of the predictors are (towards infinity).*

The combination of both hypotheses and the properties of the two distinct spatial modelling frameworks (structural and spatial dependence) defines the information horizon. For a particular dataset the information horizon is the range (or scale) above which interpretations are no longer possible, because beyond the horizon, relationships lack structure. This leads to our third hypothesis:

*Predictors with ranges of spatial dependence that are significantly longer than the range of a response variable and with low information content will fall outside of the information horizon for that property. Conversely, predictors with ranges or scales that are similar or shorter than that of a response are interpretable, assuming that there is a relevant structural relationship to the soil or environmental property.*

Therefore, to build interpretable models, where a feature importance analysis can return reliable results, i.e. where the descriptive accuracy is high, the following is required:

- predictors with relevant structural relationships to the response, and
- predictors with ranges of spatial dependence that are not much longer than the range of the response variable.

Here, we develop a method to examine under which conditions obviously meaningless predictors can result in accurate model validations. We also investigate whether an information horizon can be derived and which problems one might encounter when trying to interpret predictors that fall beyond the information horizon. Finally, we introduce a new measure to describe the information content of spatial predictors.

## Methods

### Study sites

**Figure 1:**
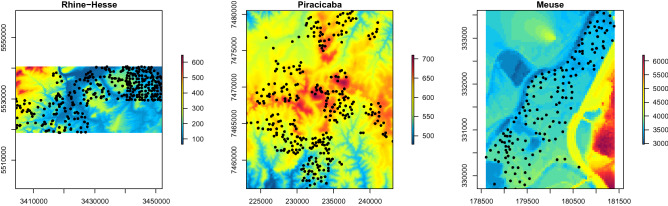
Digital elevation models and location of the sample sites of the three study sites. Gaps in the Meuse DEM were closed by interpolation. Units are in meters for Rhine-Hesse and Piracicaba and in centimeters for Meuse.

The description of the study sites is reproduced form Behrens et al.^4^. Figure 1 shows the sample locations draped over the corresponding digital elevation models (DEM).

The Meuse dataset consists of 155 samples of the River Meuse floodplain in the Netherlands^8,9^. The heavy metal distribution across the floodplain is driven by polluted sediments carried by the river Meuse and mostly deposited close to the river bank and areas with lower elevation. In this study log-transformed zinc concentration was used. The resolution of the digital elevation model (DEM) is 40 m.

The Rhine-Hesse (Germany) data set is an example of a dataset with a strongly autocorrelated distribution of soil properties. Samples of the top-soil silt content (n = 342) of the 0–10 cm depth interval were available. The silt content is based on wind erosion and material translocation from the Rhine-Main lowlands to the surrounding heights of Rhine-Hesse. The resolution of the DEM is 20 m.

The Piracicaba study area comprises a sugarcane growing region in Brazil. Soil samples (n = 321) of topsoil clay content (0–10 cm) were used for modelling. Soil formation patterns reflect those of the rock formations, strike and dip and subsequent erosion due to a relatively high precipitation. The clay content was transformed using sqrt(clay). A SRTM DEM with a resolution of 90 m was used.

### Adjustable meaningless predictors

To test the first two hypotheses, we created and tested artificial predictors, which have no structural relationship to the response variable, but where we can adjust the spatial dependence of the model. Therefore, we use Gaussian random fields (GRF)^10^ based on the spherical predictor model (see “Variography” section), where we can set the range of the spatial dependence to a particular value and thus can test the relation to the range of a response variable. To generate the GRFs we used the geoR package^11^ in R^12^.

**Figure 2:**
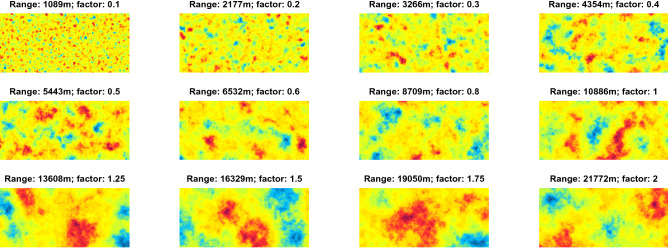
Examples of Gaussian random fields with different variogram ranges for the Rhine-Hesse study site. A factor of 1 corresponds to the range of the soil property.

To systematically test the effect of different variogram ranges of the GRF predictors on prediction accuracy, we used the following multipliers for the variogram ranges of the soil properties: 0.1, 0.2, 0.3, 0.4, 0.5, 0.75, 1.0, 1.25, 1.5, 1,75, 2.0, 2.5, 3.0, 3.5, 4.0, 4.5 and 5.0. If a resulting range was greater than the diagonal distance of the study area, we ignored it. One realization of a GRF for the first 12 ranges for the Rhine-Hesse study area is shown in Fig. 2. For each range we generated 100 independent GRFs and used them as predictors in machine learning models where a soil property was set as the dependent response variable.

From the second hypothesis, we can assume that the longer the range of a predictor is beyond the range of the response variable, the more similar it is to an EDF and therefore modelling requires fewer predictors. To test this, we also built models based on only 10 randomly selected GRFs.

To prevent additional random effects, we denoised the GRFs, which are generally very noisy compared to the corresponding DEM and interpolations of the soil property. Hence, we set the nugget to zero and filtered each GRF with a simple blur filter.

### Variography

To calculate the range of spatial dependence of a soil property, we derived spherical variograms using the gstat package^13^ in R^12^. The spherical model has the most interpretable values for nugget, sill and range, as it does not approach the sill asymptotically and has a true range. This was important in our work as we needed to automatically analyze and compare different datasets^4^.

Commonly in gstat, the range is initialized to a cutoff value which is 1/3rd of the diagonal length of the study area. Since, we are interested in analyzing the effects of multiscale modelling, we set the cutoff distance to the full diagonal of the study area.

Fitting the variograms automatically, will not always produce good estimates for the range^4^. Therefore, we estimated the range using a Loess smoother^14^ on the empirical variogram and then selected the first maximum of the resulting curve as the estimate of the range. This provides a good basis for comparing the ranges of the different datasets.

**Figure 3:**
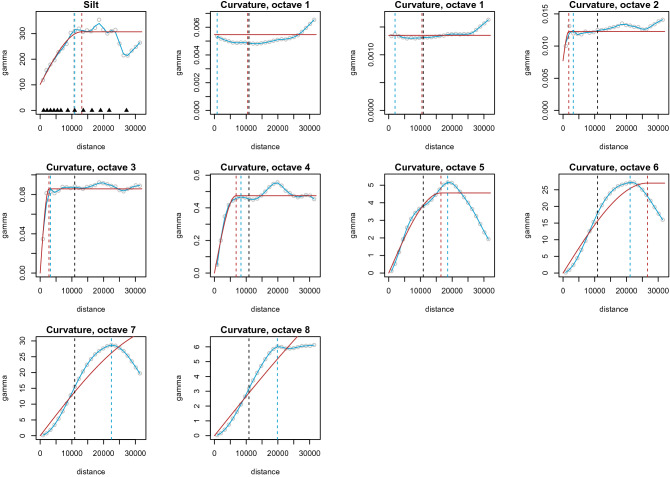
Variograms for the silt content and multi-scale versions of mean curvature in Rhine-Hesse. Red: spherical variogram and range; blue: Loess regression and range; black: soil property range. The triangles in the first variogram represent the ranges of the Gaussian random field tested in this study.

We applied the same approach to estimate the ranges of the predictors, which is based on a random sample set of 10,000 points across the square area around the sample location. Figure 3 shows variograms for topsoil silt content and for curvature across all scales of the Rhine-Hesse dataset where a spherical variogram is compared to the results based on the Loess smoother.

### Information content

In some cases, the variogram range of a predictor is not sufficient to assess its interpretability with relation to a specific scale and the size of the geographical sample space. For example, elevation often has a long variogram range and it would be removed from the set of interpretable predictors if we used only the soil property range as a criterion for assessing predictor interpretability. However, elevation often has an influence on the distribution of environmental properties and it can also show large local variations despite its long variogram range. Additionally, if the variogram range of a response variable is relatively short with relation to the size of the study area, it is possible that predictors with ranges that are longer than the range of the response are interpretable, especially in cases of non-stationarity.

**Figure 4:**
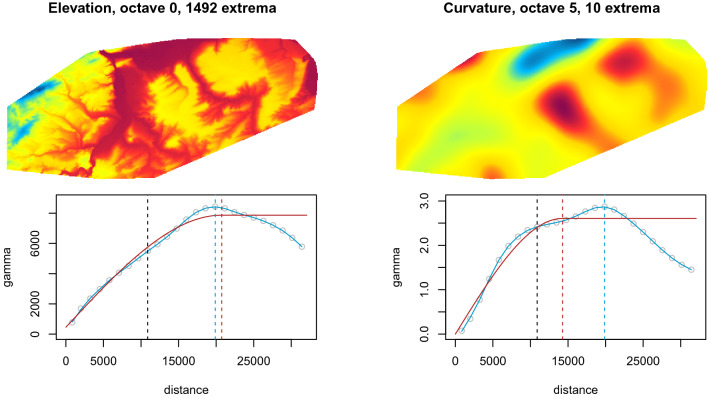
Predictors with high ranges but relevant information content for the Rhine-Hesse study site. The extrema are counted within the convex hull around the sample locations. Red: spherical variogram and range; blue: Loess regression and range; black: soil property range.

Therefore, we implemented another indicator of information content, where we count the number of local maxima and minima of a predictor within the convex hull area around the sample locations. If there would be only two local extrema, the predictor should have similar variogram properties as EDFs. However, if the number of extrema is above a threshold, we should be able to visually interpret it. In this study we set the number of local extrema threshold to 10. Figure 4 shows examples of two predictors with large variogram ranges but relevant information content for the relevant convex hull area.

### Reference models

The benchmark model to test structural dependence used Gaussian mixed scaling (GMS)^15^ of relevant terrain attributes. Gaussian mixed scaling is an approach to decompose scales of numerical environmental predictors and specifically terrain attributes. It is based on the Gaussian pyramid^16^, which in each step reduces the cell size by half. In mixed scaling, the terrain attributes are derived based on downsampled versions of the DEM. The terrain attributes are up-scaled back to the original resolution of the DEM. The maximum possible number of scales, also called octaves, was set to 10 and the minimum number of pixels for rows and columns was set to 4^17^.

**Figure 5:**
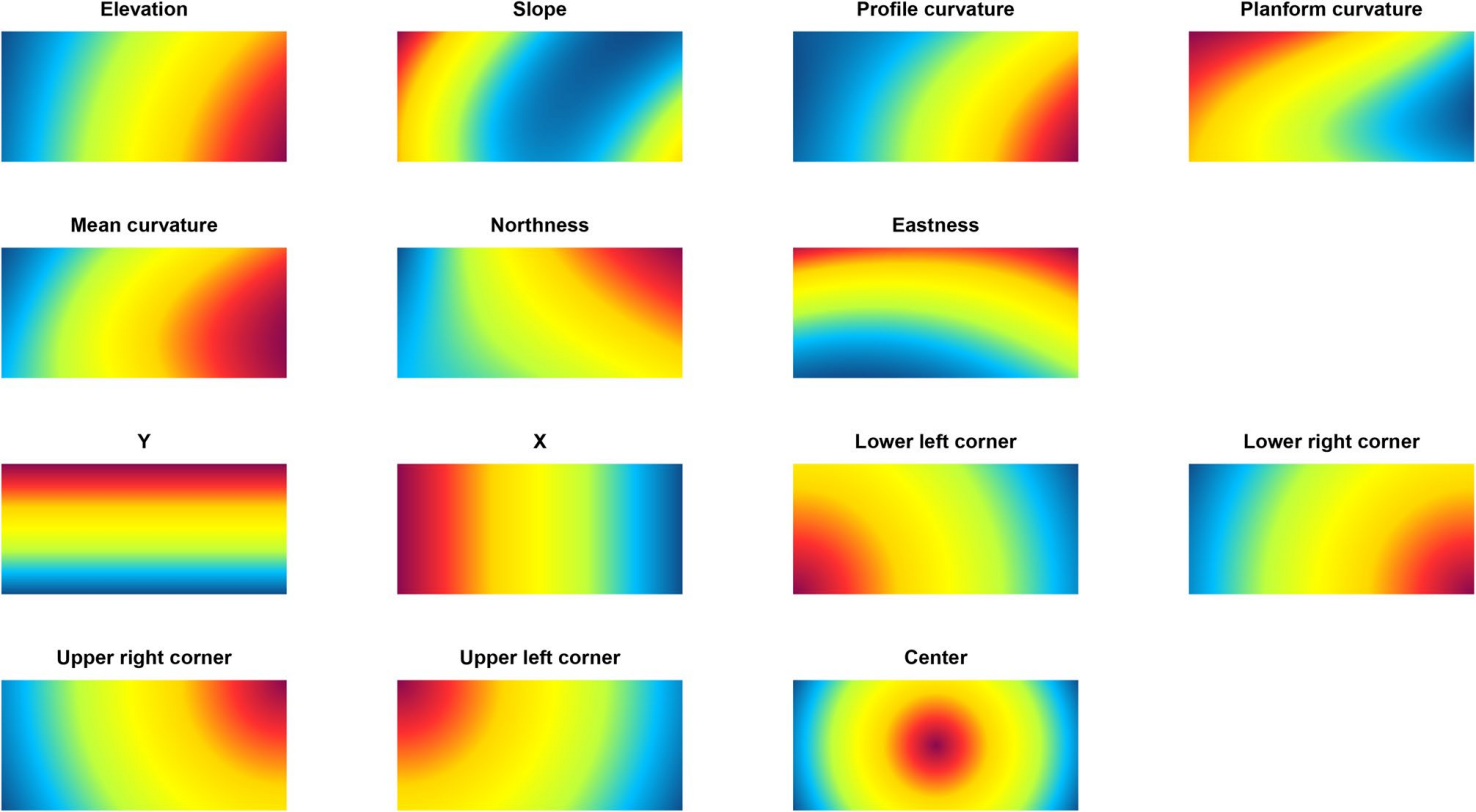
Comparison between the coarsest terrain scales of the Gaussian mixed scaling approach (rows 1 and 2) and the Euclidean distance fields (rows 3 and 4) for the Rhine-Hesse dataset.

The benchmark model to test spatial dependence, used EDFs as predictors^5^. Apart from the X and Y coordinates, as in trend surface analysis^18^, five additional EDF predictors were used in this approach. These are the distances to each corner of a rectangle around the sample locations as well as the distance to the center of the study area. The EDF predictors for the Rhine-Hesse dataset are shown in Fig. 5.

Since it is possible that the coarsest GMS scales resemble the EDF predictors (with infinite ranges; Fig. 5), we additionally tested a GMS model in which we removed all terrain predictors with very long ranges. The aim was to produce a (restricted but) interpretable reference model.

To estimate the effect of non-stationarities we also tested a model in which we merged the restricted GMS dataset with the EDF dataset. We assumed that if this model leads to higher predictive accuracies compared to the restricted GMS model, spatial dependencies are present that cannot be interpreted on the basis of the available structurally relevant predictors.

### Machine learning and validation

We used Random Forests (RF^19,20^ as the machine learning model in R^12^ with 2000 trees. Caret^21^ was used for grid learning to optimize the number of predictors tested in each split of a tree in the forest, and to validate the models with cross-validation. We used tenfold cross-validation with random assignments, which is one of the most widely used validation approaches. To provide stable results we used 10 repeats and averaged the respective validation accuracies. For a detailed discussion the interested reader is referred to^22–24^.

## Results and discussion

### Meaningless predictors and spatial dependence

**Figure 6:**
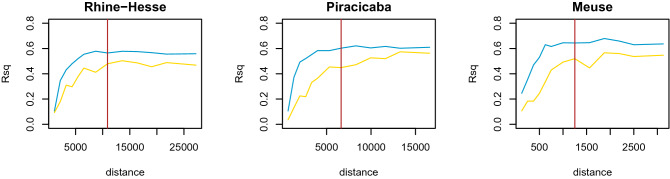
Influence of the variogram ranges of Gaussian Random Fields (GRF) on predictive accuracy. Blue: $R^2$ values for models based on 100 GRFs; gold: $R^2$ values for models based on 10 GRFs; red: variogram range of the corresponding soil property.

The variogram range of the GRF predictors has a strong influence on model validations. Figure 6 shows the increase in the cross-validation $R^2$ with increasing ranges for all three study sites. The maximum prediction accuracy is reached when the range of the structurally meaningless predictors (e.g. the GRFs) is similar to the range of the soil properties. This is also the reason why meaningless predictors, with respect to a structural relationship to a soil property, such as photographs of faces or paintings, can produce accurate evaluation statistics.

The effect is more stable when 100 GRFs are used, compared to only 10 GRFs for each range of spatial dependence. That is, when using 100 GRFs, relatively accurate model validations are already reached using lower scale predictors compared to using only 10 GRFs. However, cross-validation accuracy can be relatively high when the variogram ranges of the 10 GRFs are long and thus better resemble the effects of EDFs.

We can therefore accept the first two hypotheses: using enough but totally meaningless predictors with similar or longer ranges of spatial dependence than that of the response variable, can result in models that produce high predictive accuracies, however, with zero descriptive accuracy. Both, GRFs and EDFs are spatial but not environmental predictors. So, we can “interpolate” with only a few but very large-scale random predictors. But when we interpolate, we cannot interpret. And when the predictors are not completely linear as EDFs, which is the case for GRFs with random meaningless variations between the sample locations, smoothing between the sample points, which is the concept behind interpolation, is not guaranteed. Hence, using GRFs or otherwise meaningless predictors is neither a structural model nor an interpolation model in the classical sense, and therefore must be rejected in principle.

### Reference models and prediction accuracy

**Figure 7:**
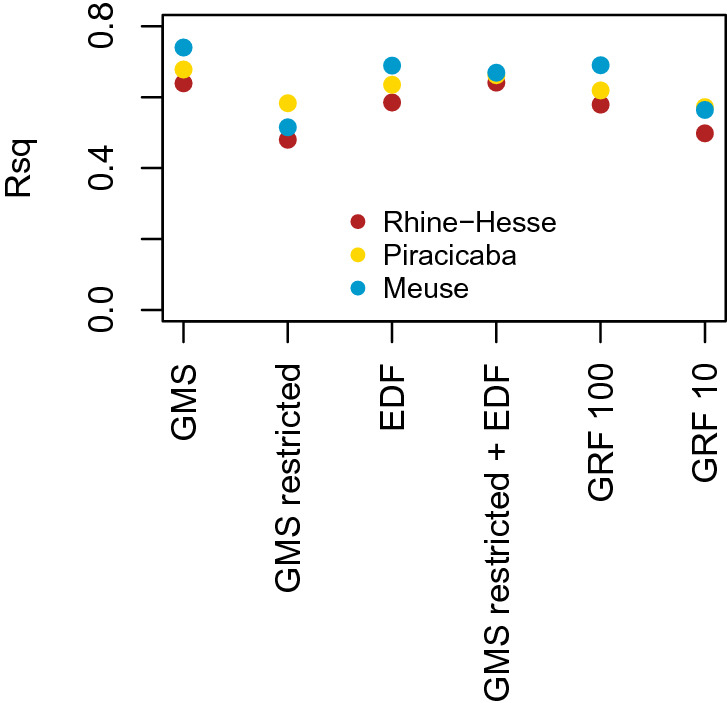
Modelling cross-validation accuracies (represented by the coefficient of determination, $R^2$) for all approaches and datasets (GMS: Gaussian mixed scaling, EDF: Euclidean distance fields, GRF: Gaussian random fields models with 100 or 10 predictors).

**Figure 8:**
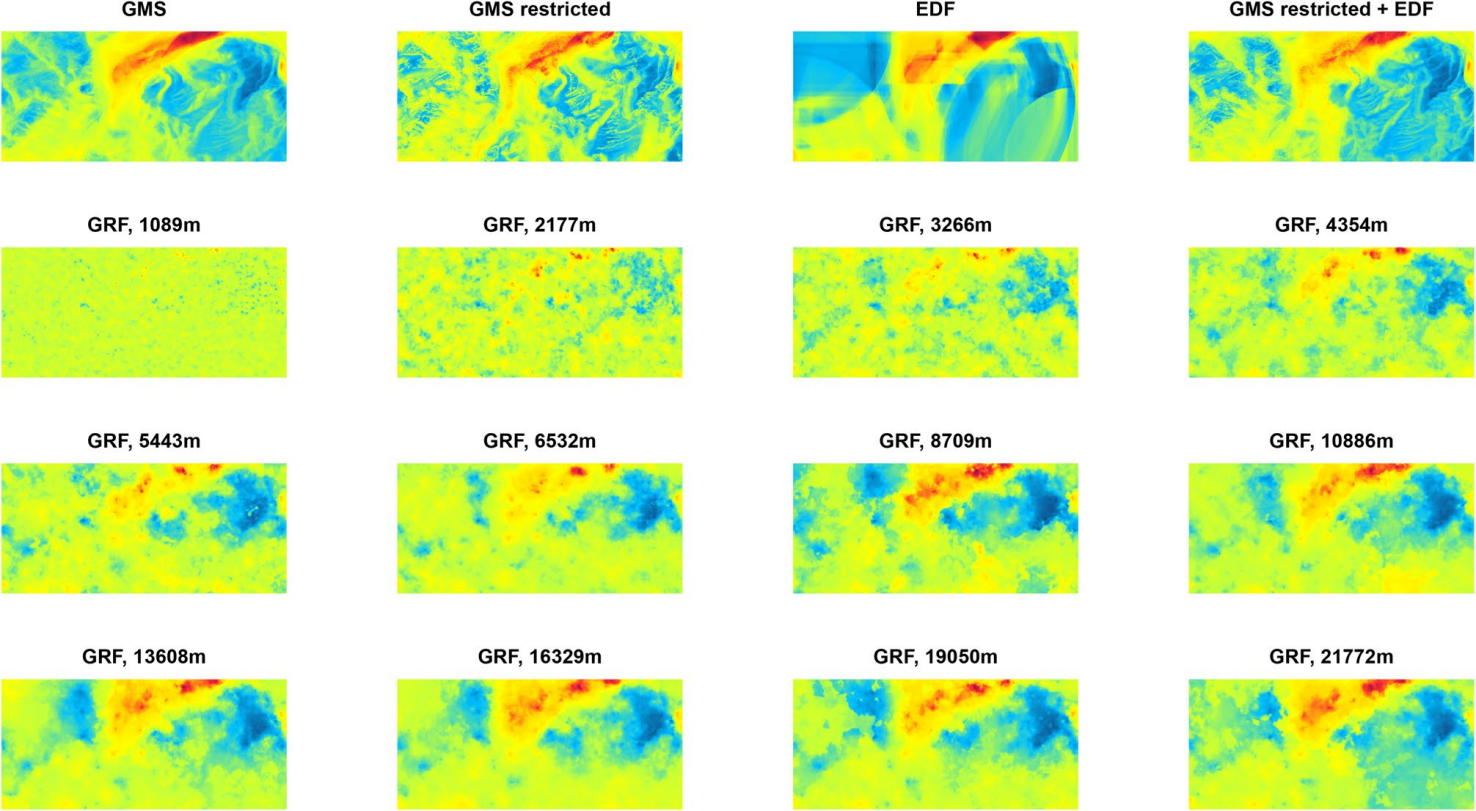
Modelling results. The first row shows the reference models (GMS: Gaussian mixed scaling, EDF: Euclidean distance fields). The other three rows show the models based on 100 Gaussian random fields (GRF) with different variogram ranges.

The prediction accuracies of the different models are presented in Fig. 7. Figure 8 shows the corresponding maps.

Generally, the GMS models produced the best results. This could be an effect of the number and structure of the predictors, which is larger compared to the merged restricted GMS + EDF dataset, and thus possibly an effect of overfitting to the dependence structure of the data^23^.

Models with EDFs generally performed better than models with the restricted GMS. Hence, the spatial dependence of soil properties cannot be described by the constrained GMS dataset. There is an increase in prediction accuracy when using the EDFs together with the restricted GMS dataset (except for the Meuse dataset), which could be an indicator for non-stationarity.

In terms of prediction accuracy, modelling with GRFs produced relatively high predictive accuracies (Fig. 7).

### The information horizon—descriptive accuracy and relevance

Generally, the descriptive accuracy is strong if there is a causal relationship^25^ between the response and a predictor. The descriptive accuracy can also be high if there are associations among variables as usually inferred by statistical analysis, which can suggest potential causal relationships^1^.

Provided that the relevance of the predictors is given and the algorithm or method to generate the data is valid^15, 17^, the results of this study indicate that the descriptive accuracy is high if the range of spatial dependence of a predictor is equal or smaller than the range of the response variable. However, predictive accuracy can increase when predictors with longer ranges than the range of the environmental property are included, possibly due to non-stationarities in the environmental process^4,26^ or effects of anisotropy. Therefore, one should remove only those predictors from multiscale approaches^15,17,27–30^ with variogram ranges that are long with respect to the size of the study area and if their information content is below a certain minimum (Fig. 4).

If, on the other hand, predictors show ranges of about the diagonal length of the entire study area, then they resemble the properties of the EDFs (Fig. 5) and their ranges are too long to lend themselves to interpretation. In these cases, predictors behave indistinguishably from purely spatial predictors, although they might still be interpretable in some situations.

In summary, these results confirm our third hypothesis, and show that the primary information horizon is located somewhere between the range of the variogram of the soil property and a certain minimal variation of the predictors across the study site.

### Beyond the information horizon—descriptive uncertainty and contextual complexity

We recently showed that when finer to coarser scales are successively removed from a set of all scales of a GMS modelling, prediction accuracy usually remains high, even if only the coarsest scales remain in the model^4^. In cases where the prediction accuracy decreases, we can assume that (i) structural information is lacking, that (ii) interpolation is not the appropriate method, or that (iii) the spatial dependence of the coarse scale GMS predictors are not suitable for interpolation, e.g. if all these coarse scale predictors only show a trend in one direction, for example in X direction only instead of X and Y direction.

We also found an increase in prediction accuracy beyond the range of the variogram of soil properties in GMS and similar approaches^4,15,26^, when successively adding coarser scales. There are two explanations. First, not all original terrain properties show exactly the same original scale or range, which is due to the convolution functions and the general approaches to calculate terrain properties (e.g. first and second order derivatives, i.e. slope and curvature). Second, this effect might be related to non-stationarity, where coarse-scale predictors can help to “divide” the study area into zones. We tested this here by combining the restricted set of GMS predictors, which are within the information horizon, with EDFs. In all cases prediction accuracy increased. Hence, there is obviously some spatial dependence present, resulting from predictor interactions on very coarse scales or long ranges, which are beyond the information horizon, inferable by the size of the study area. In these cases, there will be some uncertainty in the descriptive accuracy when using predictors that show information contents below a certain minimum of spatial variation.

Looking specifically at the three study sites we see complex soil property formation processes due to interactions of predictors at different scales. This contextual complexity has to be taken into account when interpreting environmental predictors beyond the information horizon, as discussed above.

In Piracicaba very coarse-scale predictors are important^27^. The soil formation system, however, is rather simple. It is based on rock formation, strike and dip, and subsequent erosion. In this case coarse scale terrain indicators for aspect are good proxies to differentiate between the two different types of parent material, even though they resemble properties of EDFs. In such cases partial dependence models should be applied to aide interpretation^27^.

The silt content in Rhine-Hesse is controlled by local silt translocation^31^, which occurred in the last glacial period of the Pleistocene epoch (Würm glaciation) and which was modulated by interactions of climate and terrain. This can be described in terms of a teleconnected system^32^ and can be mapped by terrain only, which then serves as a proxy for that system^26,27^. Similar to Piracicaba, interpretations of predictors with very large ranges and relatively low information content can be reasonable. However, the descriptive uncertainty is higher compared to predictors that fall within the information horizon.

The situation for Meuse is different due to a different dominant process system. The zinc content is driven by flooding events. Therefore, different and more relevant predictors, such as the distance to the river Meuse, should be used in this case^9^. We see that EDFs perform better compared to the mixed dataset (GMS restricted + EDF). This shows that the multiscale terrain predictors are not relevant, but represent noise, and can therefore serve at most as vague proxies. Another problem resulting in such an effect could be algorithmic issues related to feature selection within the Random Forests model, which in some specific cases might occur in relation to autocorrelated predictors^33^, or to effects due to fitting noise^5^.

Interestingly, in all cases the GMS models perform better compared to the mixed dataset (GMS restricted + EDF). This can be either due to a higher number of predictors in the GMS approach or relevant structural predictors beyond the information horizon.

Generally, the interpretation of environmental predictors beyond the edge of the information horizon needs specific care and is afflicted with more uncertainty.

## Conclusions

We investigated interpretable machine learning. We showed that there is a strong relationship between the range of spatial dependence of a soil property, and the range of spatial dependence of predictors, even if the predictors are meaningless. We also showed that predictors with long ranges, including those that are meaningless, behave like predictors with infinite ranges, such as EDFs, which are unassociated to the pedogenic formation of the soil property. We defined the information horizon, which relates the range of spatial dependence, described by the variogram range, to the information content of the predictors. When a relevant set of predictors are used, it ensures accurate and interpretable modelling. Finally, using three study sites, we demonstrated the conditions under which predictors that fall beyond the information horizon can be interpreted. Since correlation is not causality, the following principles apply:

- for models to be interpretable, only domain-relevant and structurally related predictors must be used;
- the scales of the predictors must be kept within the range of spatial dependence of the soil or other environmental variable and their information content in the study area should be interpretable;
- structural and spatial dependence should be analyzed separately;
- interpretations of potentially relevant predictors that fall beyond the information horizon should be made with care.

The violation of these principles contradicts domain knowledge and understanding. Following them, and ensuring that they have a similar feature space coverage does not only allow interpretations of the models but potentially also their transfer. As well as issues such as overfitting and controlling confounding effects, contextual complexity at coarse scales is one of the greatest challenges for interpretable spatial machine learning.

## Data Availability

The Meuse data set that supports the findings of this study is available through the R package sp^9^. The other datasets were used under license for the current study, and thus are not publicly available. Data are however available from the corresponding author upon request depending on the permission of the licensors.
